# Management of gastrointestinal stromal tumours in the Imatinib era: a surgeon's perspective

**DOI:** 10.1186/1477-7819-6-77

**Published:** 2008-07-18

**Authors:** Ravindra S Date, Nicholas A Stylianides, Kishore G Pursnani, Jeremy B Ward, Muntzer M Mughal

**Affiliations:** 1 Department of Gastrointestinal Surgery, Lancashire Teaching Hospital NHS, Foundation Trust, Preston Road, Chorley, Lancashire, PR7 1PP, UK

## Abstract

**Background:**

Surgical resection has remained the mainstay of treatment of GIST with a 5-year-survival of 28–35%. Tyrosine kinase inhibitor (Imatinib) has revolutionised the treatment of these tumours. The current research is directed towards expanding the role of this drug in the treatment of GIST. We present our experience of managing GIST in this institute.

**Methods:**

This is a case note study of patients identified from a prospectively kept database from January 2000 to August 2007.

**Results:**

16 patients were diagnosed with GIST. The median age was 66 years (range 46 to 82) and the male to female ratio was 9:7. Eleven patients underwent surgery, 9 of which had R0 resection (2 laparoscopic, 1 converted to open), one had an open biopsy and one had a debulking procedure. 3 patients were inoperable and 2 were found to be unfit for surgery. Five patients received Imatinib (2 postoperatively). The risk assessment based on morphological criteria showed that 4 patients had low, 4 had intermediate and 8 had high malignant potential. The median follow up was for 12 months (range 3–72); 2 patients died of unrelated causes at 6 and 9 months after diagnosis.

**Conclusion:**

Most GISTs can be managed effectively using existing protocols. However currently there is no evidence based guidance available on the management of GIST in the following situations-role of debulking surgery, the follow up of benign tumours not requiring surgical resection and role of laparoscopic surgery. Further research is needed to answer these questions.

## Background

Gastrointestinal stromal tumours (GIST) represent a subgroup of mesenchymal tumours, which were traditionally known as leiomyomas or leiomyosarcomas and have traditionally been treated by surgery. The results of a simple surgical resection with clear margins were comparable to those of a radical resection [1]. Therefore until recently simple resectional surgery remained the mainstay of treatment with 5-year-survival rates of 28–35%[2,3] for R0 resections. Introduction of Imatinib mesylate (tyrosine kinase inhibitor) for the treatment of GIST at the beginning of this century has improved outcomes in metastatic and unresectable tumours. Demetri et al have shown that Imatinib is useful in the treatment of unresectable or metastatic GIST with more than half the patients showing a sustained response [4]. Verweij et al have shown that a dose of 400 mg twice a day achieves significantly longer progression-free survival [5]. Two small retrospective studies have suggested that neoadjuvant Imatinib therapy may have a role in advanced GIST and have suggested a prospective evaluation [6,7]. Most of the current research is directed towards establishing the role of Imatinib as an adjuvant to surgery [8-11]. Management of small GIST is mainly in the form of watchful waiting as suggested in the consensus statement by ESMO[12], however there is no strong evidence to support this statement.

In spite of these developments the role of surgery itself has remained unchanged. We present our experience of 16 cases managed in this institute since introduction of Imatinib.

## Methods

A case note study of all the patients diagnosed with GIST from January 2000 to August 2007 was carried out. Cases were identified from a prospectively kept database in the unit.

## Results

16 patients were diagnosed with GIST during the study period. The demographics, presentation, histology, management and follow up of these patients are summarised in Table 1. Eleven patients underwent surgery, 9 of which had R0 resection. Two of these patients had laparoscopic wedge excision and in one laparoscopic operation had to be converted to open to ensure R0 resection. One patient had an open biopsy to confirm the diagnosis before commencing Imatinib. The median follow up was for 12 months (range 3–72). Two patients died of unrelated causes at 6 and 9 months after the diagnosis.

**Table 1:** Summary of patients with GIST

**Patient**	**Age, Gender**	**Site**	**Presentation**	**Maximum diameter (mm)**	**CD117 & CD34**	**Mitosis per HPF**	**Operation**	**Imatinib**	**Follow up (months)**	**Risk**
1	66, F	Stomach	Mass	70	Positive	10/50	Wedge resection	No	5, SD	High
2	82, M	stomach	GI bleed	60	Positive	2/50	Wedge resection	No	12, SD	Inter
3	60, M	Stomach	GI bleed	75	Positive	2/50	Lap to open Wedge resection	No	12, SD	Inter
4	72, M	Stomach	Pain and distension	110	Positive	300/50	Debulking	Yes	22, SD	High
5	51, M	Stomach	Mass	14.5	Positive	None seen	Inoperable*	Yes	3, PD	High
6	61, F	?Stomach/?extra-gasttrointestinal	Mass and distension	260	Positive	8/50	Excision and total hysterectomy	Yes *#*	45, SD	High
7	72, M	Stomach	Mass	90	Positive	17/50	Inoperable**	Yes	6, Died of MI	High
8	68, F	Stomach	GI bleed	70	Positive	4/50	Lap. wedge resection	No	24, SD	Inter
9	70, F	Oesophagus	Dysphagia	20	N/A	N/A	Not fit	No	29, SD	N/A
10	46, F	Stomach	GI bleed	70	Positive	10/50	Distal gastrectomy	No	3 < SD	High
11	77, F	Stomach	GI bleed	80	Positive	34/50	Distal gastrectomy	No	9, Died	High
12	47, M	Stomach	GI bleed	50	Not done	Not done	Wedge resection	No	72, SD	N/A
13	60, M	Duodenum	Incidental	13	N/A	N/A	Not fit	No	14, SD	N/A
14	74, F	Stomach	GI bleed	40	Positive	2/50	Lap. wedge resection	No	9, SD	Low
15	57, M	Duodenum	Cholangitis	40	Positive Negative	None	Duodenectomy	No	62, SD	Low
16	76, M	Stomach	GI bleed	65	Negative	2/50	No	Yes	7, SD	Inter

N/A: No histological diagnosis available

*: Metastatic disease

**: locally advanced

*#*: Imatinib was commenced 2 years after the operation when patient was found to have recurrence.

SD: static disease at last follow up.

PD: progressive disease at last follow up.

Patient 6 was operated by Gynaecologist for fibroids and intraoperatively surgeons were called to remove a large irregular mass adherent to the greater curvature of the stomach and infiltrating the omentum. There was no evidence of peritoneal or liver metastasis. The mass was removed completely which proved to be GIST on histology with possible extra-gastrointestinal or gastric in origin.

Patients 9 and 13 had GIST in the oesophagus and the second part of the duodenum respectively. Due to their associated co-morbidities they were deemed unfit for major resectional surgery. In both the cases endoscopic biopsies were insufficient to give the histological diagnosis, but EUS (endoscopic ultrasound) findings were consistent with the diagnosis of GIST. They were both followed up by yearly EUS examinations.

Patient 16 had significant iscaemic heart disease and could not withstand staging laparoscopy and hence the operation is deferred till cardiac status improves.

## Discussion

This cohort of patients showed a significant variation in presentation and wide range of disease stages at presentation. Most of these patients could be managed effectively with surgery and/or Imatinib using current protocols. Imatinib was used in patients with unresectable or metastatic disease according to the NICE guidelines[13].

Our experience suggests that current guidelines are clear for the management of unresectable and metastatic GIST; however surgeons are faced with management dilemmas in the following situations.

### • Small GIST

Small GISTs are often diagnosed radiologically as an incidental finding (patient 13 in our series). They are labelled as "benign" purely on the basis of their size and radiological appearance. These tumours, particularly those located in the oesophagus or the duodenum, are difficult to biopsy and in the absence of histological diagnosis there is a potential risk of keeping a malignant GIST under observation or exposing benign GIST to unnecessary surgery. This unnecessary surgery could mean a pancreaticoduodenectomy in such a patient or an oesophagectomy in patient 9, if deemed fit for operation. Currently there is no guidance available on the rationale of regular follow up or of the use of Imatinib in these patients. There are few reported series of endoscopic enucleation of these tumours [14,15]. However this is not a widely accepted practice due to lack of robust evidence.

Many patients with a small GIST and requiring regular follow up do not get reported leading to a lack of data regarding long-term survival. There is a need for a central database of these cases to improve reporting and long-term follow-up.

### • Debulking surgery

At the other end of the spectrum are locally advanced tumours (such as patient 4 in our series). This patient clearly benefited from "debulking" surgery followed by Imatinib. He underwent debulking surgery due to pressure symptoms even in the presence of peritoneal metastasis. This approach, which would have been seen as "unconventional" then, proved to be beneficial to the patient. This supports the current view of using Imatinib with cytoreductive surgery for synchronous metastasis [16]. Currently there are number of reports showing increased recurrence-free and overall survival after surgery for metastatic GIST following TKI therapy [17-19]. However, these reports were largely comprised of patients with recurrent disease *after *the initial resection of the primary disease, and none specifically focused on the role of primary debulking surgery.

The role of surgery may need redefining in such patients.

### • Adjuvant therapy with Imatinib

There are reports showing benefits of adjuvant Imatinib in GIST of high malignant potential [9]. Current trials (ACOSOG Z9000, ACOSOG Z9001, EORTC and SSG XVIII) are addressing the role of adjuvant Imatinib in different groups of patients. The interim results of ACOSOG Z9001 suggest that Imatinib increases recurrence-free survival when administered following the complete resection of a primary GIST. Our number-6 patient could have potentially benefited from such therapy. She had an excision of a large tumour found incidentally by the gynaecologists. This proved to be GIST of high malignant potential on histology. Adjuvant treatment was not administered, as she had an R0 resection. She remained disease free for 2 years but subsequently developed peritoneal metastasis, which were then treated with Imatinib. We believe that the treatment of such patients should be more aggressive with the use of adjuvant Imatinib while waiting for the final outcomes of ongoing trials.

### • Laparoscopic surgery

The change in nomenclature from leiomyosarcoma to GIST and the advent of Imatinib has changed the general perception of these tumours to be of benign nature, and there is an increasing trend towards laparoscopic treatment [20,21]. It is evident from current literature that R0 resections give the best chance of long term cure to such patients and that tumour spillage along with an R1/R2 resection is associated with an increased incidence of recurrence [22]. We feel that GIST should be treated following the principles of "cancer surgery" and that open resection with adequate margins should be performed unless an R0 resection is achievable laparoscopically.

Due to limited number of patients we have not applied any statistics to the data but tried to highlight the grey areas in the management of these patients.

## Conclusion

We feel that future surgical trials need to be directed towards

• Long-term follow up of small GIST located in areas that are not readily accesible and diagnosed soley on *radiological *findings and are presumed to be *benign*.

• Defining the role of debulking surgery, with or without down staging of disease.

Laparoscopic surgery should be considered only if it is not compromising the principles of cancer surgery.

## Competing interests

The authors declare that they have no competing interests.

## Authors' contributions

Mr Stylianides helped in acquisition of data and preparation of the first draft. Mr Date was responsible for conception of idea, overall preparation and revision of the manuscript. Mr Mughal, Mr Pursnani and Mr Ward were responsible for management of the patient and revising the manuscript critically for important intellectual content. All authors read and approved the final manuscript.
